# A Challenging Case of Oral Ulcers and Gastrointestinal Bleeding: Crohn's or Behçet's Disease

**DOI:** 10.1155/2023/4705638

**Published:** 2023-10-04

**Authors:** Marina A. S. Dantas, Ana Luiza Graneiro, Rodrigo Cavalcante, Lina Maria Felipez

**Affiliations:** ^1^Department of Pediatric Hospital Medicine, Medical University of South Carolina, Charleston, SC, USA; ^2^Department of Allergy and Immunology, Nicklaus Children's Hospital, Miami, FL, USA; ^3^Department of Cardiology, Children's Hospital of Philadelphia, Philadelphia, PA, USA; ^4^Department of Gastroenterology, Nicklaus Children's Hospital, Miami, FL, USA

## Abstract

*Introduction*. Differentiating Crohn's disease (CD) and Behçet's disease (BD) with gastrointestinal (GI) manifestations can be clinically challenging, as current diagnostic criteria are not clear between both conditions and multiple symptoms could overlap. *Case Presentation*. The patient is an 8-year-old boy of Brazilian descent, who initially presented with a 1-year history of painful oral ulcers. Before presenting to the hospital, he had been treated for periodic fever, aphthous stomatitis, pharyngitis, and adenitis and placed on steroids, with relapsing symptoms on attempts to wean the doses. The initial workup was largely unremarkable. Buccal biopsies showed no granulomas, and the ophthalmologic exam was normal. Infectious and rheumatological tests were negative. Prometheus IBD sgi testing showed a pattern consistent with CD; however, the patient had multiple negative endoscopies, colonoscopies, and capsule endoscopies. He developed intermittent bloody stools and severe malnutrition and did not respond to infliximab, colchicine, or methotrexate. After a large GI bleed, a 4th colonoscopy was performed, which showed large round ulcers in the terminal ileum, and no granulomas. He was started on ustekinumab with clinical improvement. One month later, he developed bilateral hip effusion and meningismus, being diagnosed with aseptic meningitis secondary to COVID-19. He improved, but in one month developed worsening symptoms, and MRV showed extensive venous sinus thrombosis. The patient was started on enoxaparin, methylprednisolone, and colchicine, with resolution of the thrombus on a 3-month follow-up. The patient's overall symptoms remained controlled with clinical and biochemical remission on monthly ustekinumab. *Discussion and Conclusion*. Our patient had a challenging clinical course, with nonspecific systemic and intestinal manifestations which proved difficult to differentiate between BD and CD. Given endoscopic findings and the worsening of an auto-inflammatory reaction in the central nervous system after COVID-19 in a patient with controlled GI symptoms, the most likely diagnosis is BD.

## 1. Introduction

**Figure 1:**
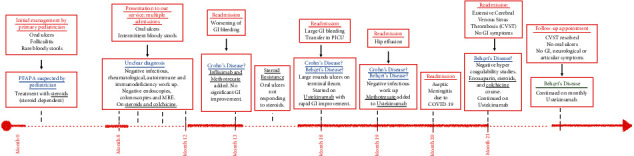
Patient's symptoms timeline. COVID-19: severe acute respiratory syndrome coronavirus 2 (SARS-CoV-2); CVST: cerebral venous sinus thrombosis; PFAPA: periodic fever, aphthous stomatitis, pharyngitis, and adenitis; GI: gastrointestinal; PICU: pediatric intensive care unit.

Differentiating Crohn's disease (CD) and Behçet's disease (BD) with gastrointestinal (GI) manifestations can be clinically challenging. Although BD is a systemic vasculitis, it can present with symptoms mimicking CD, including mucocutaneous lesions, involvement of the GI tract, and episodic arthritis [1, 2]. Since current diagnostic criteria are not clear between both conditions and multiple symptoms could overlap, a thorough investigation of end-organ involvement and immunohistological findings may help to specify the diagnosis. We present a case of intricate mimicry, illuminating subtle nuances between CD and BD that played a pivotal role in our diagnostic process (Figure 1).

## 2. Case Presentation

The patient is an 8-year-old boy of Brazilian descent with a history of autism, who initially presented to our hospital with an 8-month history of painful oral ulcers. He had a one-time history of a perianal ulcer and folliculitis on his scalp and buttocks, as well as intermittent fever and bloody stools. He had no history of genital ulcers. He had been treated for PFAPA (periodic fever, aphthous stomatitis, pharyngitis, and adenitis) and placed on steroids by his primary pediatrician, with relapsing symptoms on attempts to wean the doses. Family history was noncontributory. His initial physical exam was significant for multiple large oral ulcers involving his tongue, palate, and lips. He exhibited neither abdominal tenderness nor masses, perianal ulcers, signs of arthritis, nor any other dermatological manifestations. Differential diagnosis was broad including infectious causes, rheumatological and immunodeficiencies. The initial workup was largely unremarkable, including tests for systemic lupus erythematosus, herpes simplex virus, Epstein–Barr virus, tuberculosis, cyclic neutropenia, primary immunodeficiencies, and periodic fever syndromes (Table 1). He had a negative ophthalmological exam and no pathergy. Buccal biopsies showed chronic inflammatory findings with granulation tissue, no granulomas, and positive findings for *Fusobacterium periodontium*. Prometheus IBD sgi testing showed a pattern consistent with CD: positive anti-A4-Fa2 IgG, anti-Fax IgG, ATG 10L1 (heterozygous), NKX2-3 (heterozygous), ICAM-1, SAA 179, and elevated CRP. ASCA and ANCA were negative. However, the patient had multiple negative endoscopies/colonoscopies with ileal intubation. We utilized a negative capsule endoscopy to exclude the possibility of small bowel involvement, as double-balloon enteroscopy was not accessible within our pediatric institution. He also had multiple abdominal images including CT of the abdomen and MRI enterography showing no abnormalities.

Despite being on steroids and colchicine 0.6 mg twice a day, he developed worsening bloody stools and severe malnutrition (BMI *z*-score −2.38) around 13 months from the beginning of his symptoms. At that point, he was transferred from the rheumatology service to the GI service, due to growing suspicions of CD vs. BD. Infliximab (IFX) 10 mg/kg (weeks 0, 2, and 6) and methotrexate (MTX) 12.5 mg once a week were started. After 12 weeks of biologic initiation, he did not exhibit improvement. After being on high-dose steroids (30 mg/day) for 6 months, his previously responsive mouth ulcers did not subside. Since the IFX level was 10.5 µg/mL and 0 antibodies, the decision to schedule IFX 10 mg/kg every 4 weeks was made.

**Figure 2:**
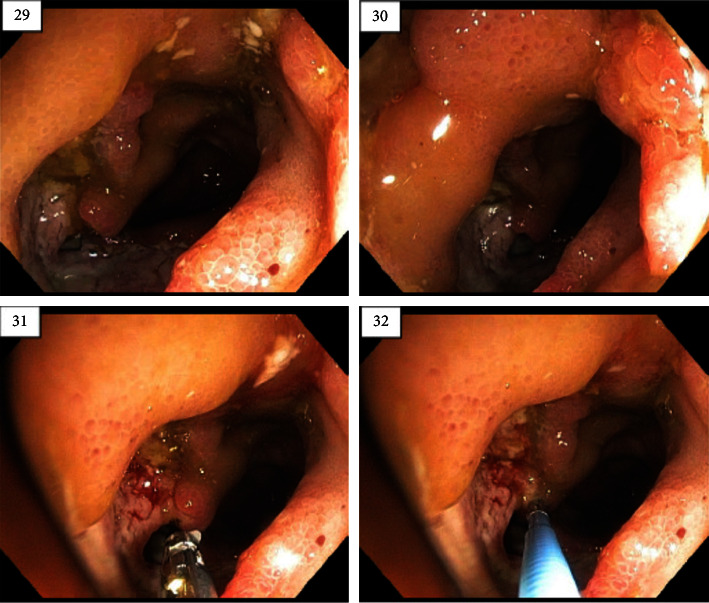
Patient's 4th colonoscopy findings showing large ulcer.

At month 18, he developed a large GI bleed. A 4th colonoscopy was performed (Figure 2), which showed large round ulcers in the terminal ileum, with granulation tissue, primarily chronic inflammatory infiltrates, no granulomas, and no cobblestoning, a pattern that was not specific for CD, but did not rule out the condition. He was treated as an anti-TNF nonresponder and started on ustekinumab (UST) 390 mg IV followed by 90 mg SQ doses every 4 weeks with clinical improvement after the first dose. UST induction level was 25 *µ*g/mL and 0 antibodies.

Despite the improvement of the GI symptoms, the patient presented with bilateral hip effusion at month 19. MTX 10 mg once a week was added with a suboptimal response. Synovial fluid culture and 16S bacterial DNA were negative. He subsequently developed meningismus, and CSF studies were compatible with aseptic meningitis secondary to COVID-19. Brain MRI showed no significant findings and improved with supportive care.

**Figure 3:**
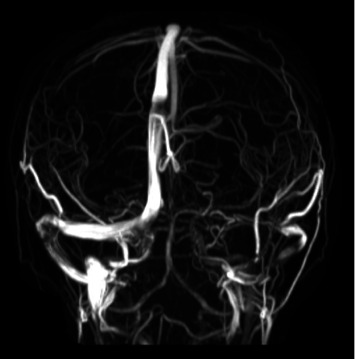
Patient's extensive cerebral venous sinus thrombosis on magnetic resonance venography (MRV).

He returned to the hospital within a month, with worsening headaches, emesis, and fevers. Repeat CSF studies were negative for infectious processes but showed a high opening pressure of 39 cm H_2_O. MRI, MRA, and MRV showed extensive sinus thrombosis of the superior sagittal sinus, straight sinus, and proximal aspect of the right transverse sinus (Figure 3). A hypercoagulability panel was negative. The decision was made to decrease inflammation and treat it as Behçet's disease since gastrointestinal symptoms had improved after UST, which decreased the likelihood of CD as the underlying cause. The patient was started on enoxaparin 1 mg/kg twice a day, methylprednisolone 2 mg/kg a day, and colchicine 0.6 mg twice a day, with the resolution of the thrombus on a 3-month follow-up visit. The patient's neurological symptoms subsided and overall symptoms remained controlled with clinical and biochemical remission on monthly UST.

## 3. Discussion and Conclusion

Our patient had a challenging clinical course, with nonspecific systemic inflammatory findings and intestinal involvement, which can be seen in both CD and BD. Although intestinal manifestations are more classically seen in CD, 10–15% of patients with BD can have gastrointestinal involvement. There are other clinical conditions that could present with a similar constellation of symptoms, such as intestinal tuberculosis, but these were ruled out. Other shared signs and symptoms between CD and BD include uveitis, oral ulcers, pyoderma gangrenous, peripheral arthritis, and thrombotic events. There are no pathognomonic tests or laboratory findings to differentiate both conditions, and the final diagnosis depends on the patient's clinical progression and on the weighted interpretation of multiple clinical signs and parameters [1, 2].  Table 2 shows the most common manifestation in each condition, highlighting those present in the case in discussion.

From an endoscopic standpoint, our patient had multiple negative colonoscopies, but one finally revealed significant disease with round big ulcers in the terminal ileum, without granulomas or cobblestone appearance. This pattern aligns more closely with BD than with CD based on the current literature. A retrospective observational study conducted by Lee et al. showed that round ulcer shape was the most specific independent factor to discriminate BD from CD (*p* < 0.001), while the absence of cobblestone appearance was the most sensitive independent factor (*p*=0.044) [3].

Although cerebral venous sinus thrombosis is a recognized thrombotic complication of both CD and BD, it is more common in the latter, especially in the presence of neurological symptoms (Neuro-Behçet's disease). In BD, neurological symptoms may be secondary to vascular events, primary parenchymal involvement (meningitis and encephalitis), or longitudinal transverse myelitis [1–5]. In addition, despite COVID-19 being reported as a cause of thromboembolic complications, the severity of our patient's manifestation suggests the existence of an underlying inflammatory disorder inducing a prothrombotic state, rather than an isolated postinfectious manifestation of COVID-19 [6].

Given the endoscopic findings and worsening of an auto-inflammatory reaction in the central nervous system in a patient with controlled GI symptoms, we concluded that the most likely diagnosis was BD. Even though the patient had a prometheus IBD sgi testing showing a pattern possibly positive for CD, he had a negative ASCA-IgA/IgG and pANCA, which contribute the most to the entire predictive value of the 17-marker test [7]. Interestingly, while the treatment of both BD and CD is similar, with the use of steroids, immunomodulators, and biologic agents, our patient was resistant to multiple medications, only presenting improvement of oral ulcers upon initiation of UST, a drug with promising results for the treatment of refractory mucocutaneous BD [8].

The presented case may help physicians in the diagnosis and treatment of intestinal BD, which has an overall worse prognosis than CD. This is particularly important as the immigration of Asian and Mediterranean populations increases in the United States, as these groups have a higher prevalence of BD and may contribute to it being more commonly seen in the country [9].

## Figures and Tables

**Table 1:** Initial laboratory work-up on presentation to our service.

Laboratory test	Value
WBC	11.1 × 10^9^ cells/L
Hemoglobin	12.6 g/dL
Platelets	303 × 10^9^ cells/L
ESR	12 mm/hr
CRP	<0.5 mg/L
ALT	37 U/L
AST	50 U/L
Albumin	4.3 g/dL
INR 1.13	1.13
Stool calprotectin	**90.9 µg/g**
Zinc level	Within normal limits
Selenium level	Within normal limits
Lymphocyte subsets	Within normal limits
Complement levels	Within normal limits
Immunoglobulins (IgG, IgA, IgM, and IgE)	Within normal limits
RAST testing for common food allergens	Negative
Lupus panel	Negative
Prometheus test	**Compatible with CD**
HLA B27	Negative
HLA B51	Negative
Period fever syndromes genetic panel	Negative
Celiac panel	Negative
CMV IgM	Negative
EBV panel	Negative
Hepatitis panel	Negative
HSV PCR	Negative
HIV antibodies	Negative
QuantiFERON	Within normal limits

WBC: white blood cell count; ESR: erythrocyte sedimentation rate, CRP: C-reactive protein, ALT: alanine aminotransferase, AST: aspartate aminotransferase, INR: international normalized ratio, RAST testing: radioallergosorbent test, HLA: human leukocyte antigen, CMV: cytomegalovirus, HSV: herpes simplex virus, HIV: human immunodeficiency virus. Bold values represent abnormal values.

**Table 2:** Similar and different characteristics of Behçet's disease and Crohn's disease.

Clinical history	Crohn's disease	Both	Behçet's disease
Demographics	More common in Northern Europe, Northern America, United Kingdom. Female > male		Asian and Mediterranean populations. Male > female

Genetic and laboratory markers	NOD2/CARD15, ASCA, ANCA		HLA-B51

Ophthalmologic	Mostly insidious onset. Bilateral anterior uveitis, dry eyes, blepharitis, and episcleritis	Uveitis	More recurrent and sight-threatening. Pan-uveitis or posterior uveitis with necrotizing retinal vasculitis

Oral		**Oral aphthous ulcers**	

Cardiovascular		**Thrombotic events**	Superficial thrombophlebitis, **venous thrombosis**, arterial aneurysm. Pulmonary artery thrombosis

Gastrointestinal	On endoscopy, discontinuous longitudinal ulcers (≥4 to 5 cm), cobblestone appearance, and/or small aphthous ulcerations are arranged in a longitudinal fashion. Noncaseating granulomas, cryptic architectural abnormalities, and discontinuous inflammation. Anal strictures, fistula, and abscess formation	**Gastrointestinal symptoms**	**On endoscopy, single or few (≤5), large (>1 cm), discrete, and round or oval-shaped ulcerations in the ileocecal area.** Other forms include neutrophilic phlebitis and ischemia damage (vasculitis)

Hepatobiliary	Primary sclerosing cholangitis		Budd-Chiari syndrome

Genitourinary			Genital ulcers with scar, epididymitis/orchitis

Dermatological	Pyoderma gangrenous, sweet's syndrome, neutrophilic dermatosis	Erythema nodosum	Pathergy, pseudofolliculitis

Musculoskeletal		**Peripheral arthritis,** sacroiliitis, spondyloarthropathy	

Neurological			**Neuro-Behçet**

Patient's manifestations are in bold. The table has been adapted from the works of Yazisis [1] and Valenti et al. [2].

## Data Availability

All data underlying the results are available as part of the article, and no additional source data are required.

## Abbreviations

ALT: Alanine aminotransferase
AST: Aspartate aminotransferase
BD: Behçet's disease
BMI: Body mass index
CD: Crohn's disease
CMV: Cytomegalovirus
COVID-19: Severe acute respiratory syndrome coronavirus 2 (SARS-CoV-2)
CRP: C-reactive protein
CSF: Cerebrospinal fluid
CVST: Cerebral venous sinus thrombosis
EBV: Epstein–Barr virus
ESR: Erythrocyte sedimentation rate
GI: Gastrointestinal
HLA: Human leukocyte antigen
HSV: Herpes simplex virus
IBD: Inflammatory bowel disease
IFX: Infliximab
INR: International normalized ratio
MRA: Magnetic resonance angiography
MRI: Magnetic resonance imaging
MRV: Magnetic resonance venography
MTX: Methotrexate
PFAPA: Periodic fever, aphthous stomatitis, pharyngitis, and adenitis
PICU: Pediatric intensive care unit
RAST testing: Radioallergosorbent test
SLE: Systemic lupus erythematous
TNF: Tumor necrosis factor
UST: Ustekinumab
WBC: White blood cell count.
